# The phosphatidylinositol 3-phosphate effector FYVE3 regulates FYVE2-dependent autophagy in *Arabidopsis thaliana*

**DOI:** 10.3389/fpls.2023.1160162

**Published:** 2023-03-15

**Authors:** Jeong Hun Kim, Hyera Jung, Kyoungjun Song, Han Nim Lee, Taijoon Chung

**Affiliations:** Department of Biological Sciences, Pusan National University, Busan, Republic of Korea

**Keywords:** ATG8, autophagosome, autophagy, phosphatidylinositol 3-phosphate, phosphoinositide, vacuolar trafficking

## Abstract

Phosphatidylinositol 3-phosphate (PI3P) is a signaling phospholipid that play a key role in endomembrane trafficking, specifically autophagy and endosomal trafficking. However, the mechanisms underlying the contribution of PI3P downstream effectors to plant autophagy remain unknown. Known PI3P effectors for autophagy in *Arabidopsis thaliana* include ATG18A (Autophagy-related 18A) and FYVE2 (Fab1p, YOTB, Vac1p, and EEA1 2), which are implicated in autophagosome biogenesis. Here, we report that FYVE3, a paralog of plant-specific FYVE2, plays a role in FYVE2-dependent autophagy. Using yeast two-hybrid and bimolecular fluorescence complementation assays, we determined that the FYVE3 protein was associated with autophagic machinery containing ATG18A and FYVE2, by interacting with ATG8 isoforms. The FYVE3 protein was transported to the vacuole, and the vacuolar delivery of FYVE3 relies on PI3P biosynthesis and the canonical autophagic machinery. Whereas the *fyve3* mutation alone barely affects autophagic flux, it suppresses defective autophagy in *fyve2* mutants. Based on the molecular genetics and cell biological data, we propose that FYVE3 specifically regulates FYVE2-dependent autophagy.

## Introduction

Endomembrane trafficking comprises dynamic membrane flow where various membrane lipids and cargo proteins are sorted and transported to their appropriate destinations. Newly synthesized proteins are targeted to various endomembrane compartments *via* multiple routes, including secretory and vacuolar trafficking. Proteins endocytosed from the cell surface are sorted at the endosomal compartments and either recycled to the plasma membrane or targeted to the vacuole (or lysosome in animals) for degradation. Cytoplasmic proteins may be sequestered by the autophagic membrane and delivered to the vacuole/lysosome for degradation.

The best-characterized type of autophagy is macroautophagy (hereafter referred to as autophagy), in which a membrane sac called the phagophore is expanded and sealed to form the autophagosome sequestering a small portion of the cytoplasm (Marshall and Vierstra, 2018). The autophagosome fuses with the vacuole, releasing its cargo into the vacuolar lumen as an autophagic body. Finally, the autophagic body is rapidly degraded by acid hydrolases in the vacuole. The formation of phagophores and autophagosomes is facilitated by autophagy-related (ATG) proteins, which are conserved in nearly all eukaryotes. ATG8 is a ubiquitin-fold protein conjugated to the membrane lipid phosphatidylethanolamine on the phagophore. As ATG8 resides on the autophagic membrane even after autophagosome fusion, lipidated ATG8 is a useful marker for phagophores, autophagosomes, and autophagic bodies. ATG5 and ATG7 are among the ATG proteins essential for the ATG8 conjugation and general autophagy, whereas ATG2 and ATG18A form a protein complex that transfers membrane lipids to the phagophore and contributes to phagophore growth (Kim et al., 2020).

Phosphatidylinositol 3-phosphate (PI3P) is a signaling phospholipid that plays regulatory roles in endomembrane trafficking (Schink et al., 2013). In eukaryotic cells, PI3P is enriched at the cytosolic leaflet of endosomal, autophagosomal, and vacuolar (or lysosomal) membranes. In budding yeast and plants, PI3P is produced from phosphatidylinositol by the phosphatidylinositol 3-kinase (PI3K) complex containing the catalytic subunit VPS34 (Vacuolar Protein Sorting 34). There are two distinct PI3K complexes which have 3 common and 1 specific subunits. The PI3K complex I, consisting of VPS34, VPS15, VPS30/ATG6, and ATG14, is responsible for PI3P production at the autophagic membrane, while the PI3K complex II, having VPS38 instead of ATG14, generates PI3P on the endosomal membrane (Schink et al., 2013).

Despite its low abundance in biological membranes, PI3P on endosomal and vacuolar membranes is essential for plant development. Null mutations of *vps34* and *atg6* in *Arabidopsis thaliana* cause lethality (Fujiki et al., 2007; Lee et al., 2008), and *vps38* mutants show pleiotropic phenotypes resulting from defective endosomal sorting and vacuolar trafficking (Lee et al., 2018; Liu et al., 2018; Yuan et al., 2018). Less obvious developmental phenotypes are observed in *Arabidopsis atg14a atg14b* double mutants, although they are defective in autophagy (Liu et al., 2020). Generally, plants with compromised functions of vacuoles and endosomes display lethality or severe developmental abnormality (González Solís et al., 2022), whereas autophagy-deficient plants do not. Instead, the autophagy mutants are hypersensitive to different types of abiotic and biotic stress (Qi et al., 2021).

The mechanism underlying the contribution of PI3P to autophagy and endosome trafficking remains poorly understood in plant cells (Chung, 2019). The regulatory roles of PI3P in endomembrane trafficking are primarily mediated by PI3P effectors, which bind to PI3P and, directly or indirectly *via* protein interactions, serve different downstream functions such as cargo selection, membrane bending, vesicle transport, tethering, scaffolding, and signaling (Schink et al., 2013). In *Arabidopsis*, PI3P-binding proteins have been shown to localize at endosomal and/or autophagic membranes. For example, SNX1 (Pourcher et al., 2010) and SNX2b (Phan et al., 2008) localize to endosomes, and FYVE4 (Liu et al., 2021) is primarily cytosolic but can be recruited to endosomal membranes. FYVE1/FREE1 (Gao et al., 2014; Gao et al., 2015), ATG18A (Zhuang et al., 2017; Kang et al., 2018; Kim et al., 2022) and FYVE2/CFS1 (Cell death-related endosomal FYVE/SYLF protein 1) (Sutipatanasomboon et al., 2017; Kim et al., 2022; Zhao et al., 2022) localize at both endosomes and autophagosomes. Genetic analyses of mutants defective in the PI3P-binding proteins indicated that these proteins are involved in endosomal sorting (e.g., SNX1, SNX2b, FYVE4), autophagic degradation (e.g., ATG18A, FYVE2/CFS1), or both (e.g., FYVE1/FREE1). However, molecular mechanisms for these putative PI3P effectors are not fully defined, and how PI3P binding impacts their functions is unknown.

PI3P effectors typically bind to PI3P *via* their FYVE, PX, or PH domains (de Jong and Munnik, 2021). In *Arabidopsis*, the FYVE domain is shared by FYVE1/FREE1, FYVE2/CFS1, FYVE3/CFS2, and FYVE4. Both FYVE2/CFS1 and FYVE3/CFS2 proteins harbor the PI3P-binding FYVE domain and C-terminal SYLF domain, which assists yeast SYLF-containing proteins to bind actin filaments and phosphoinositides (Robertson et al., 2009; Urbanek et al., 2015; Sutipatanasomboon et al., 2017) (**Figure S1A**). FYVE2/CFS1 facilitates the efficient progression of autophagy and interacts with a variety of proteins such as ATG18A, SAR1B, and VPS23A, which are important for autophagosome biogenesis, COPII vesicle trafficking, and membrane scission, respectively (Sutipatanasomboon et al., 2017; Kim et al., 2022; Zhao et al., 2022). By contrast, no apparent defect in autophagic flux was observed in its paralogous *fyve3/cfs2* single mutants (Kim et al., 2022; Zhao et al., 2022). Thus, currently no evidence supports the involvement of FYVE3/CFS2 in autophagy and other membrane trafficking routes.

Here, we aimed to assess genetic and cell biology data to ascertain the role of *Arabidopsis* FYVE3/CFS2 in autophagy. We showed that FYVE3 was incorporated into the autophagic machinery *via* interaction with ATG8. An autophagy pathway consisting of core ATG proteins and PI3P effectors was proposed, based on our genetic data indicating a specific involvement of FYVE3 in FYVE2-dependent autophagy.

## Materials and methods

### Plant materials

The *Arabidopsis thaliana* T-DNA insertional mutants of *atg2-1* (Inoue et al., 2006)*, atg5-1* (Thompson et al., 2005), *atg7-2* (Chung et al., 2010), *fyve2-2*, and *fyve3-1* (Kim et al., 2022) and transgenic plants expressing *ProUBQ10:GFP-ATG8A* (Kim et al., 2013) and *ProVHA-a1:VHAa1-GFP* (Dettmer et al., 2006) were previously described, and their progenies were used*. ProUBQ10:YFP-ARA7, ProUBQ10:YFP-SYP32* (Geldner et al., 2009), and *ProUBQ10:Citrine-2xFYVE* (Simon et al., 2014) were obtained from the Arabidopsis Biological Resource Center (ABRC).

### Clones and DNA constructs

cDNA and expression clones were obtained from the ABRC with the following identifiers: *Arabidopsis ATG6* (G19464), *FYVE2* (G18828), *FYVE3* (GC105091), SA*R1B* (G19464), *SAR1C* (G11541), pIX-Halo-ATG8F (HALO_SFI_23-A01), pIX-Halo-ATG8I (HALO_SFI_71-H06), PSAT4-DEST-n(1-174)EYFP-C1 (CD3-1089), PSAT5-DEST-c(175-END)EYFP-C1(B) (CD3-1097). To make entry clones containing FYVE3 cDNA lacking the stop codon and TRAF1A, *FYVE3* and *TRAF1A* cDNA were amplified using primers listed in **Supplementary Table 1**. The *FYVE3* and *TRAF1A* PCR products were inserted into the pENTR/TOPO and pDONR221 vectors (Thermo Fisher Scientific), respectively. cDNA entry clones containing ATG8E, ATG8F, ATG8I, and ATG18A was generated as described previously (Kim et al., 2022).

### Preparation of transgenic plants

To obtain binary vectors for transgenic plants expressing *ProUBQ10:GFP-FYVE3* or *ProUBQ10:mCherry-FYVE3*, recombination of the entry clone containing *FYVE3* cDNA with *pMDC99-AtUBQ10p-GFP* and *pMDC99-AtUBQ10p-mCherry* (Suttangkakul et al., 2011) was performed *via* the LR Clonase II reaction (Thermo Fisher Scientific). The binary vectors were introduced into *Agrobacterium tumefaciens* strain GV3101. To obtain stable transformants, Agrobacterium transformants were used to infect *Arabidopsis* using the floral dip method (Clough and Bent, 1998).

### Plant growth conditions

Seeds were surface-sterilized with 50%(v/v) bleach and then washed using sterile water. The seeds were germinated on 1× Murashige & Skoog (MS) solid medium [1× MS salt including vitamins (Duchefa, Haarlem, the Netherlands) with 1% (w/v) sucrose, 0.25% (w/v) phytagel (Sigma, St Louis, MO, USA)] or liquid medium with gentle shaking (100 rpm). Plants were incubated under long-day conditions (16 h light and 8 h dark) at 21-23°C.

To induce autophagy, the seedlings incubated in the liquid medium for 7 d were transferred to nitrogen-deficient medium (1 × MS micronutrient solution, 3 mM CaCl_2_, 1.5 mM MgSO_4_, 1.25 mM KH_2_PO_4_, 5 mM KCl and 1% sucrose, pH 5.7) and further incubated for indicated duration. For drug treatment, seedlings were treated with 0.5 µM AZD 8055 (LC Laboratories), 30 µM wortmannin (Sigma), and 0.5 µM Concanamycin A (Cayman).

Phenotypic analysis of the fixed carbon starvation response was performed as described (Chung et al., 2010). Briefly, after incubating seedlings on 1× MS-suc solid medium (1× MS salt including vitamins, 29 mM mannitol, and 0.25% phytagel) for 14 d, the seedlings were then deprived of light for 10 d. Seedlings resuming growth were counted after 18 d of recovery.

### Immunoblot analysis

Seedlings frozen in liquid nitrogen were homogenized using a TNPI lysis buffer [50 mM Tris-Cl, 150 mM NaCl, 0.5% (w/v) sodium dodecyl sulfate (SDS), 0.5% (v/v) Triton-X 100, 5% (v/v) glycerol, 2 mM dithiothreitol, 1 mM phenylmethyl sulfonyl fluoride, and 10 mM iodoacetamide, pH 8.0]. Lysates were clarified by centrifugation at 16,000 × *g* at 4°C for 10 min, and the supernatant was mixed with 5 × SDS- polyacrylamide gel electrophoresis (PAGE) sample buffer [200 mM Tris-HCl (pH 6.8), 25% (v/v) glycerol, 10% (w/v) SDS, 10% (v/v) 2-mercaptoethanol]. The samples were boiled at 95°C for 10 min to ensure denaturation of protein samples and loaded into SDS-PAGE. The separated proteins were then transferred onto the Immobilon-P polyvinylidene fluoride membranes (Millipore, St Louis, MO, USA).

To prepare membrane proteins, seedlings expressing *GFP-FYVE3* were homogenized with ice-chilled pestle and mortar in TNPI buffer [50 mM Tris-Cl, 150 mM NaCl, 1 mM phenylmethyl sulfonyl fluoride, 10 mM iodoacetamide, pH 8.0]. After centrifugation at 500 × *g* at 4°C for 5 min, 5% of the supernatant was set aside as total extract, and 95% of the supernatant was further separated into soluble and pellet fractions by centrifugation at 20,000 × *g* at 4°C for 15 min.

Western blot analysis was performed as previously described (Kim et al., 2022). Anti-GFP (Sigma-Aldrich 11814460001), Anti-H3 (Abcam ab1791), Anti-BiP (Agrisera AS09481), and Anti-UDP-glucose pyrophosphorylase (UGPase; Agrisera AS05 086) antibodies were used. The protein band intensity of the immunoblots was quantified by ImageJ (National Institute of Health).

### Quantitative real-time PCR analysis

To evaluate RNA abundance, seedlings were grown on 1 x MS medium for 10 d, frozen with liquid nitrogen, and homogenized in TRIsure agent (Bioline, London, UK). The RNA extract was treated with DNase I (New England Biolabs), and cDNA was synthesized using RevertAid H Minus reverse transcriptase (Thermo Fisher Scientific) with oligo(dT)_20_ primers. Quantitative real-time PCR (qRT-PCR) was performed using Step One Plus Real-Time PCR System (Applied Biosystems) and EvaGreen (SolGent, Daejeon, Korea). The relative transcript abundance of target genes was calculated by the ΔΔCT method using the *UBC9* cDNA as an internal control. All primer sequences are provided in **Table S1**.

### Yeast two-hybrid analysis

The entry clone containing FYVE3 cDNA was recombined with the destination vector pDEST22 (Thermo Fisher Scientific) *via* the LR Clonase II reaction. The resulting pDEST22-FYVE3 and pDEST32 expression vectors for bait proteins fused to SAR1B, SAR1C, ATG8A, ATG8E, ATG8F, ATG8I, ATG18A and FYVE2 (Kim et al., 2022) were used for transformation of the AH109 strain and Y187 strain through the Yeastmaker Yeast Transformation System 2 (Clontech, Mountain View, CA, USA). To determine protein interaction, yeast colonies isolated from Trp- and Leu- deficient medium were transferred to Trp-, Leu-, and His- deficient medium containing 0 and 1 mM 3-amino-1,2,4-triazole.

### Bimolecular fluorescence complementation

The expression vectors PSAT5-c(175-END)EYFP-C1(B)-SAR1B/ATG8A/ATG18A/ATG6 were described previously (Kim et al., 2022). To generate the expression vectors PSAT4-n(1-174)EYFP-C1-FYVE3 and PSAT5-c(175-END)EYFP-C1(B)-FYVE2/TRAF1A, entry clones containing FYVE3, FYVE2, and TRAF1A cDNA were recombined with the destination vectors PSAT4-n(1-174)EYFP-C1 and PSAT5-c(175-END)EYFP-C1(B), respectively, *via* the LR Clonase II reaction. The BiFC assay using *Arabidopsis* leaf protoplasts was performed as previously described (Kim et al., 2022).

### Confocal microscopy, image processing, and quantification

To observe fluorescence signals from *Arabidopsis* plants or leaf protoplasts, either an LSM 510 or AxioObserver LSM 800 confocal microscope (Carl Zeiss) was used. For LSM 510, a 488 nm laser and BP500-530IR emission filter were used to detect GFP signals. For LSM 800, a 488 nm laser was used for excitation and fluorescence was detected at the 410-546 nm range to acquire GFP, citrine, and YFP signals. To detect mRFP and mCherry, a 587 nm laser was used for excitation, and fluorescence was detected at the 595-700 nm range.

Quantitative analysis of the confocal microscopy images was conducted ImageJ (NIH) as previously described (Kim et al., 2022). Data were presented as mean ± standard error. Student’s *t*-test was used to measure significance *via* VassarStats (http://www.vassarstats.net/).

### Accession numbers

ATG2 (At3g19190), ATG5 (At5g17290), ATG6 (At3g61710), ATG7 (At5g45900), ATG8A (At4g21980), ATG8E (At2g45170), ATG8F (At4g16520), ATG8I (At3g15580), ATG18A (At3g62770), RABF2b/ARA7 (At4g19640), FYVE2/CFS1 (At3g43230), FYVE3 (At1g29800), SAR1B (At1g56330), SAR1C (At4g02080), SYP32 (At3g24350), TRAF1A (At5g43560), UBC9 (At4g27960), and VHA-a1 (At2g28520).

## Results

### FYVE3 interacts with specific ATG8 isoforms

**Figure 1:**
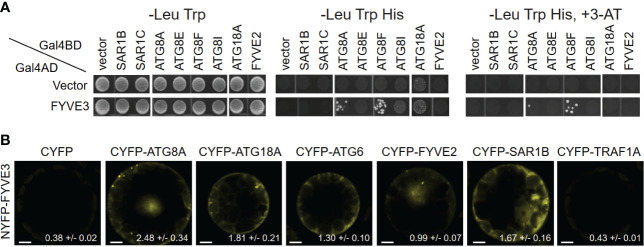
FYVE3 interacts with ATG8 and associates with FYVE2-related autophagic machinery. **(A)** Yeast two-hybrid (Y2H) interactions of FYVE3 with select ATG8 isoforms. Gal4BD, Gal4 binding domain; Gal4AD, Gal4 activation domain; 3-AT, 3-amino-1,2,4-triazole. **(B)** Bimolecular fluorescence complementation (BiFC) interactions of FYVE3 in *Arabidopsis* protoplasts. Quantification of overall reconstituted fluorescence is shown at the bottom of each image (mean ± S.E.; n = 30 to 37 images). Scale bars = 5 µm.

To gain insight into the FYVE3 protein, we studied its physical interaction with other proteins. An *Arabidopsis* interactome study identified yeast two-hybrid (Y2H) interactions of FYVE3 with ATG8D (Arabidopsis Interactome Mapping Consortium, 2011). To identify additional interactors, we performed Y2H assay using FYVE3 as a prey and select ATG proteins, FYVE2, and SAR1 isoforms as baits. Our Y2H assay demonstrated interaction of FYVE3 with select ATG8 isoforms such as ATG8A and ATG8F but not with ATG8E and ATG8I (**Figure 1A**).

The positive Y2H interactions were further tested using *in planta* interaction assays using bimolecular fluorescence complementation (BiFC) in *Arabidopsis* leaf protoplasts. The BiFC interaction of NYFP-FYVE3 with CYFP-ATG8A was verified (**Figure 1B**), whereas no considerable fluorescence was reconstituted from negative controls of NYFP-FYVE3 paired with either CYFP or CYFP-TRAF1A (Qi et al., 2017) (**Figure 1B**). In addition, interactions of FYVE3 with SAR1B, ATG18A, ATG6, and FYVE2 were detected in BiFC experiments (**Figure 1B**), although these interactions were not apparent in Y2H (**Figure 1A**). We previously showed that FYVE2 interacts with SAR1B and ATG18A (Kim et al., 2022). Thus, FYVE3 may be brought into proximity of SAR1B, FYVE2, and ATG18A, for example, by their simultaneous binding to a scaffold protein. These data suggested that FYVE3 associates with ATG8A, FYVE2, SAR1B, ATG18A, and ATG6 in plant cells.

### FYVE3 is recruited from the cytosol to autophagic and endosomal membranes

**Figure 2:**
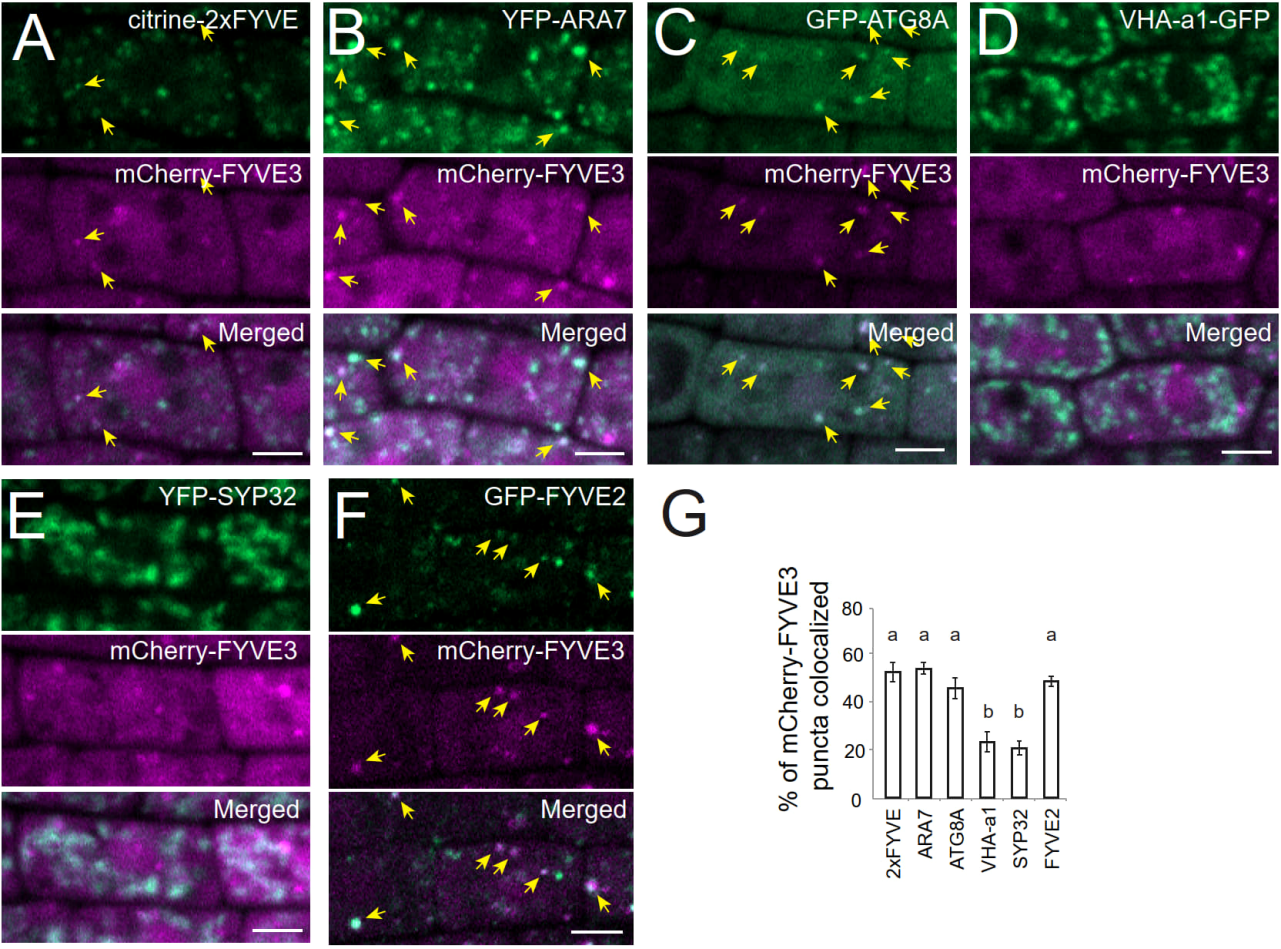
At the subcellular level, mCherry-FYVE3 is mainly cytosolic but can be found at the PI3P-enriched membrane of autophagic and endosomal organelles. **(A-F)**, Representative images of root tip cells co-expressing mCherry-FYVE3 with a variety of organelle markers, such as PI3P biosensor **(A)**, late endosome marker **(B)**, phagophore and autophagosome marker **(C)**, trans-Golgi network marker **(D)**, Golgi stack marker **(E)**, and GFP-FYVE2 **(F)**, which associates with autophagic and endosomal membranes. Scale bars = 5 µm. Arrows indicate mCherry-FYVE3 puncta that overlap with co-expressed markers, whose quantification is shown in **(G)**. Columns marked with different letters represent significantly different means according to the *t*-test (mean ± S.E.; n = 11–12 images; p < 0.01).

To investigate the subcellular distribution and trafficking of FYVE3 proteins, we prepared *Arabidopsis* transgenic plants expressing FYVE3 fused to either GFP or mCherry. The membrane fractionation experiment indicated that GFP-FYVE3 was primarily observed in the soluble fraction (**Figure S2**). We then determined the subcellular distribution of mCherry-FYVE3 by co-localizing it with various organelle markers (**Figure 2**). Root cells expressing mCherry-FYVE3 had diffuse fluorescence with relatively scarce punctate signals. The mCherry-FYVE3 puncta often overlapped with the puncta of the PI3P biosensor citrine-2xFYVE (**Figures 2A, G**), the late endosome marker YFP-ARA7 (**Figures 2B, G**), and the autophagic marker GFP-ATG8A (**Figures 2C, G**). However, relatively few mCherry-FYVE3 puncta overlapped with markers for the TGN (**Figures 2D, G**) and Golgi stacks (**Figure 2E, G**).

We also located mCherry-FYVE3 puncta relative to GFP-FYVE2 (**Figure 2F**). GFP-FYVE2 were mostly localized at cytoplasmic puncta, some of which were also decorated with mCherry-FYVE3. Although mCherry-FYVE3 puncta were fewer than GFP-FYVE2 puncta, the former mostly overlapped with the latter (**Figures 2F, G**). As FYVE2 was shown to localize on the autophagic membrane and, to a lesser extent, on the endosomal membrane (Kim et al., 2022), these data indicated that mCherry-FYVE3 puncta mainly represent either autophagic or endosomal compartments that are abundant in PI3P, whereas the diffuse signal corresponds to a cytosolic pool of mCherry-FYVE3.

### Association of FYVE3 with autophagic membranes requires ATG8 lipidation and PI3P

**Figure 3:**
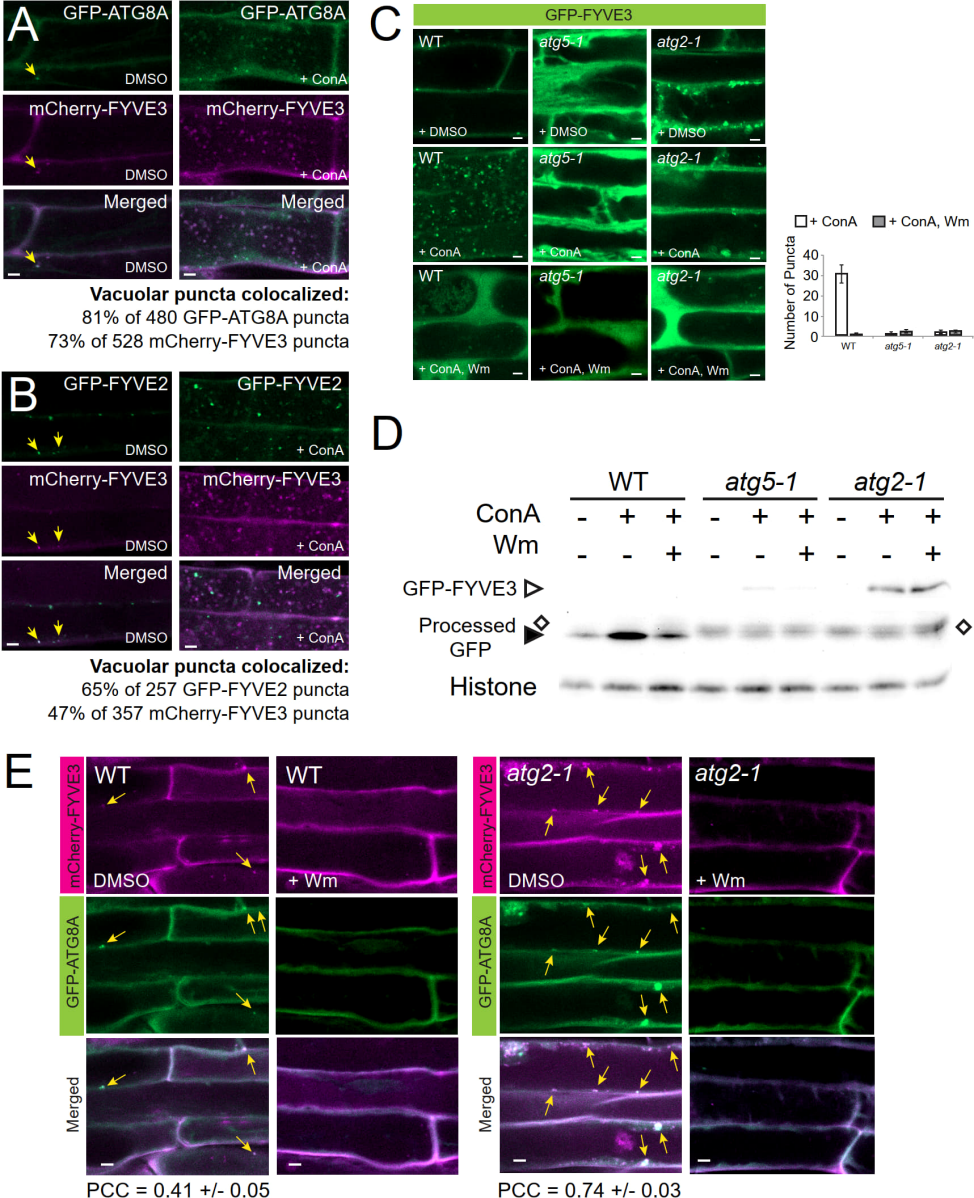
Fluorescent fusions of FYVE3 are delivered to the vacuole in a manner dependent on canonical autophagy machinery and PI 3-kinase activity. **(A)** and **(B)**, Confocal images of mature root cells co-expressing mCherry-FYVE3 with either GFP-ATG8A **(A)** or GFP-FYVE2 **(B)**. Seedlings grown in a nitrogen-rich medium were treated with DMSO or 0.5 µM ConA for 16 h prior to confocal microscopy. In the images of DMSO-treated cells, arrows indicate cytoplasmic puncta showing both GFP and mCherry signal. Percentages of vacuolar bodies showing both GFP and mCherry signal were calculated from ConA-treated cells. **(C)**, Confocal images of mature root cells expressing GFP-FYVE3 in the genetic background of WT, *atg5-1*, and *atg2-1*. Transgenic seedlings were incubated in nitrogen-rich liquid medium and treated with DMSO, 0.5 µM ConA, or 0.5 µM ConA plus 30 µM Wm for 16 h. The graph on the right shows the number of vacuolar puncta (mean ± S.E.; n = 12–13 images) per 1,000 µm^2^ area of the central vacuole. **(D)**, GFP-FYVE3 cleavage assay. WT, *atg5-1*, or *atg2-1* seedlings expressing GFP-FYVE3 were incubated as described in **(C)**, and protein extract was prepared for immunoblot analysis using anti-GFP (upper) and anti-histone H3 (lower; for loading control) antibodies. Open diamonds indicate the position of protein bands that are not stabilized by ConA and migrates slightly slower than free GFP bands (indicated by a solid arrowhead). Representative images selected from 3 repeat experiments are shown. **(E)**, Confocal images of mature root cells expressing GFP-ATG8A and mCherry-FYVE3 in WT (left images) and *atg2-1* (right images) background. Seedlings were incubated in a nitrogen-lacking liquid medium for 48 h and treated with DMSO or 30 µM Wm for 1 h prior to microscopic observation. Arrows indicate GFP-ATG8A puncta overlapping with mCherry-FYVE3 signal. The Pearson correlation coefficients (PCCs) were calculated from 10 (WT) and 13 (*atg2-1*) images (mean ± S.E.). Scale bars = 5 µm.

The subcellular distribution of FYVE3 is consistent with their protein interaction with select ATG8 isoforms, suggesting that FYVE3 binds to ATG8 on phagophores during autophagosome biogenesis. As fluorescent fusions of FYVE3 are largely co-localized with autophagic puncta, we then examined whether these proteins are delivered together to the vacuole for degradation and detected as autophagic bodies. Observation of the autophagic bodies requires the treatment of GFP-ATG8 transgenic plants with concanamycin A (ConA), an inhibitor of vacuolar proton pumps. Transgenic plants expressing both GFP-ATG8A and mCherry-FYVE3 were treated with ConA, and their root cell images were compared with DMSO-treated controls (**Figure 3A**). In ConA-treated roots, 81% of the intravacuolar mCherry-FYVE3 bodies emitted a GFP-ATG8A signal (**Figure 3A**). ConA-treated roots expressing GFP-FYVE2 and mCherry-FYVE3 also showed a moderate level of co-localization in the vacuole (**Figure 3B**). Similarly, ConA-treated root cells expressing mCherry-FYVE3 alone showed intravacuolar puncta resembling autophagic bodies (**Figure S3A**). These data indicated that fluorescent fusions of FYVE3 are degraded in the vacuolar lumen, together with ATG8A and FYVE2.

To define the molecular mechanism underlying the vacuolar trafficking of FYVE3 fluorescent fusions, we tested the effect of ConA and the PI3K inhibitor wortmannin (Wm) treatment on the subcellular localization of GFP-FYVE3 (**Figure 3C**). As expected, autophagic body-like puncta were observed in the vacuole of ConA-treated wild-type (WT) seedlings expressing GFP-FYVE3 (**Figure 3C**, WT, + ConA). Accumulation of the GFP-FYVE3 bodies was suppressed by Wm treatment (**Figure 3C**, WT, + ConA, Wm), suggesting that GFP-FYVE3 is also targeted to the vacuole *via* PI3K-dependent trafficking. To test whether core *ATG* genes are involved in this trafficking, we introgressed the *GFP-FYVE3* transgene into *atg2-1* and *atg5-1* background by a genetic cross. *atg5-1* is defective in ATG8 lipidation (Chung et al., 2010), whereas *atg2-1* accumulates a high level of lipidated ATG8 (Kang et al., 2018). When we observed their progenies by confocal microscopy, no autophagic body-like GFP-FYVE3 puncta were observed in ConA-treated *atg5-1* and *atg2-1* vacuoles (**Figure 3C**), demonstrating that canonical autophagy mediates the vacuolar targeting of GFP-FYVE3.

The vacuolar targeting of GFP-FYVE3 was validated by the immunoblot analysis using anti-GFP antibodies, similar to the GFP-ATG8 and GFP-FYVE2 cleavage assays (Kim et al., 2022). In WT, a very faint band corresponding to GFP-FYVE3 was detected, whereas free GFP released from GFP-FYVE3 was readily observed and its intensity increased by ConA (**Figure 3D**) and dark (**Figure S3B**) treatments, indicating that the free GFP band is a partial degradation product in the vacuole. In *atg5-1* and *atg2-1*, the GFP-FYVE3 band intensity was increased, whereas ConA and dark treatments did not affect the intensity of protein bands (indicated by open diamonds in **Figures 3D** and **S3B**) that migrated slightly slower than free GFP bands (solid arrowheads in **Figures 3D** and **S3B**). Combined with confocal microscopy data (**Figure 3C**), these immunoblot results indicated that GFP-FYVE3 is delivered to the vacuole for degradation *via* canonical, ATG2- and ATG5-dependent autophagy pathway. Consistently, nitrogen starvation increased the ratio of free GFP to GFP-FYVE3 in WT but not in an *atg7-2* background (**Figure S3C**). The intensity of the slower-migrating protein band appeared to increase by nitrogen starvation (**Figure S3C**). Although we do not know the nature of this protein band, the same phenomenon was observed in *atg7-2* mutants expressing the autophagy marker GFP-ATG8 (Spitzer et al., 2015). These data indicated that the vacuolar trafficking of GFP-FYVE3 showed a typical pattern of the autophagy marker.

To define the molecular requirement for the formation of GFP-FYVE3 puncta, we examined the effect of *atg5* and *atg2* mutations and Wm treatment. The abundance of GFP-FYVE3 puncta decreases and increases in *atg5* and *atg2*, respectively (**Figure S3D**). Again, Wm effectively diminished the accumulation of GFP-FYVE3 puncta in *atg2* (**Figure S3D**). The PI3P-dependent accumulation of GFP-FYVE3 puncta in *atg2* cytoplasm is very similar to that of GFP-ATG8A (Kang et al., 2018), and may result from arrested elongation/maturation of phagophores in *atg2*.

To investigate the effect of *atg2* mutation on mCherry-FYVE3 to the autophagic membrane, we co-localized mCherry-FYVE3 with GFP-ATG8A in WT and *atg2* background. *atg2* root cells over-accumulated cytoplasmic mCherry-FYVE3 puncta, which extensively overlapped with GFP-ATG8A puncta (**Figure 3E**). Thus, *ATG2* is essential for the targeting of mCherry-FYVE3 to the vacuole, but is dispensable for mCherry-FYVE3 recruitment to the phagophore.

### FYVE3 is required for the over-accumulation of autophagic structures in *fyve2* background

**Figure 4:**
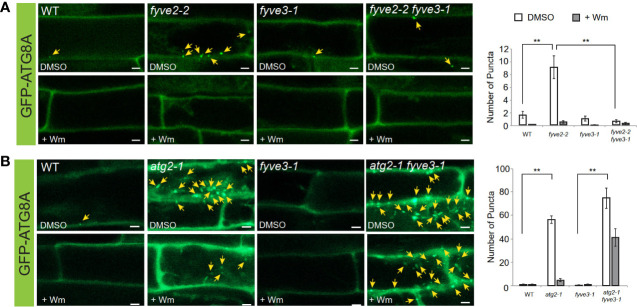
*fyve3* suppresses the accumulation of autophagic puncta in *fyve2* but not those in *atg2*. **(A, B)** Representative confocal microscopy images (left) and quantification (right) of GFP-ATG8A puncta (arrows at the images) acquired from mature root cells with indicated genetic background. Transgenic *Arabidopsis* seedlings expressing GFP-ATG8A were incubated in a nitrogen-lacking liquid media for 48 h and treated with either wortmannin (Wm) or solvent control (DMSO) for 1 h prior to image acquisition. Scale bars = 5 µm. Columns marked with asterisks represent means that significantly differ from each other, according to the *t*-test. Mean ± S.E.; n = 12 to 26 images; **p < 0.01.

To test the potential functions of *FYVE3* in autophagy, we employed *fyve3-1*, a T-DNA insertional allele (Kim et al., 2022). RT-PCR analysis indicated that *fyve3-1* is a knock-out allele, and there is no apparent transcriptional feedback between *FYVE2* and *FYVE3* genes (**Figure S1B**). Because of the FYVE and SYLF domains shared by FYVE2 and FYVE3, we initially postulated a genetic redundancy of *FYVE2* and *FYVE3*. In *fyve2* mutant background, phagophores are over-accumulated and autophagic flux is partially compromised (Kim et al., 2022). However, no significant change in autophagic flux was measured in *fyve3* single mutants, and *fyve2 fyve3* double mutants did not show further reduction in autophagic flux, when compared with *fyve2* single mutants (Kim et al., 2022; **Figure S4**). These data were inconsistent with a simple model that *FYVE2* and *FYVE3* are duplicates. To further investigate the genetic relationship between these two genes, we quantified the abundance of autophagic organelles in N-starved WT, *fyve2, fyve3*, and *fyve2 fyve3* double mutant cells expressing the autophagic marker GFP-ATG8A. Confocal microscopy analysis confirmed previous observation that *fyve2* root cells over-accumulated GFP-ATG8A puncta in the cytoplasm, compared with WT (**Figure 4A**; Kim et al., 2022). As previously reported (Kim et al., 2022), Wm effectively blocked the formation of GFP-ATG8A puncta in *fyve2* (**Figure 4A**). In contrast, *fyve3* single mutants did not accumulate GFP-ATG8A puncta (**Figure 4A**). Notably, less than 10% of GFP-ATG8A puncta were detected in *fyve2 fyve3* double mutants, relative to *fyve2* mutants (**Figure 4A**). Complementation experiments revealed that the expression of *mCherry-FYVE3* transgene in *fyve2 fyve3* mutants restored the accumulation of GFP-ATG8A puncta (**Figure S5**). These results indicate that *FYVE3* is responsible for the over-accumulation of immature autophagic structures in *fyve2*.

Based on autophagy reporter analysis using *fyve3* single and *fyve2 fyve3* double mutants, we hypothesized that FYVE3 might serve a specific function during autophagosome formation. This hypothesis was tested using a different mutant combination of *atg2* and *fyve3* (**Figure 4B**). Compared with *fyve2, atg2* mutants accumulate even more autophagic puncta, and autophagic flux is compromised almost completely (Kang et al., 2018; Kim et al., 2022). Unlike *fyve2 fyve3* double mutants, *atg2 fyve3* double mutant cells showed over-accumulation of GFP-ATG8A puncta as *atg2* single mutants did (compare **Figure 4B** with **Figure 4A**). As *fyve3* did not suppress the over-accumulation of GFP-ATG8A puncta in *atg2*, *FYVE3* appears to play a specific role in the formation of GFP-ATG8A puncta in *fyve2.* Of note, treatment with Wm only partially suppressed the GFP-ATG8A puncta in *atg2 fyve3* double mutants (**Figure 4B**).

**Figure 5:**
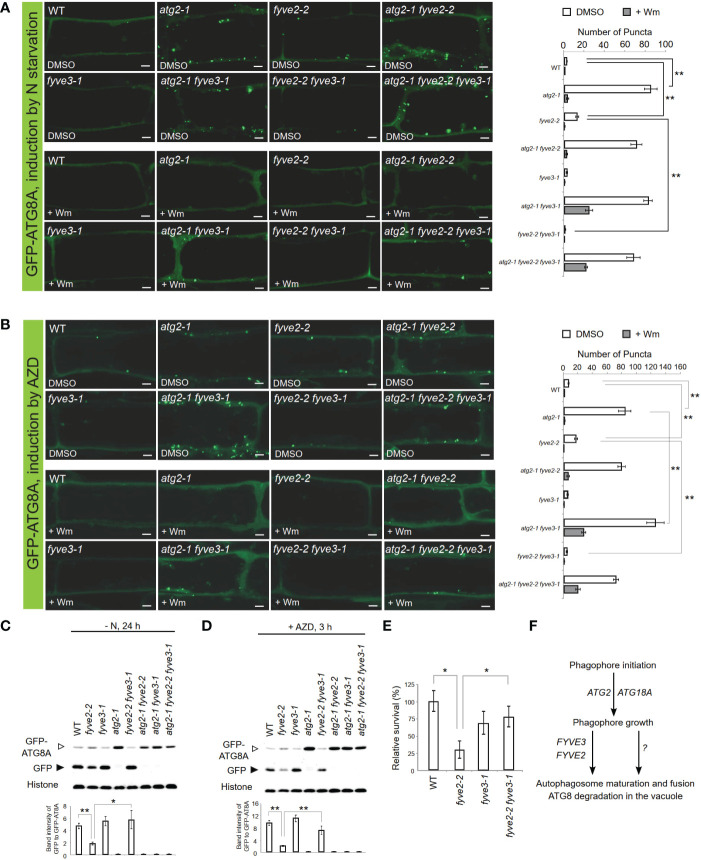
*FYVE3* acts upstream of *FYVE2* and downstream of *ATG2* in a genetic pathway defined by the autophagy marker GFP-ATG8A. **(A, B)** Representative confocal microscopy images (left) and quantification (right) of GFP-ATG8A puncta (arrows at the images) acquired from mature root cells with indicated genetic background. *Arabidopsis* transgenic seedlings expressing GFP-ATG8A were incubated in either nitrogen-lacking liquid media for 12 h **(A)** or nitrogen-sufficient liquid media containing AZD8055 for 1 h **(B)**, and treated with either wortmannin (Wm) or solvent control (DMSO) for 1 h prior to image acquisition. Scale bars = 5 µm. **(C, D)**, GFP-ATG8A cleavage immunoblot assays. *Arabidopsis* transgenic seedlings expressing GFP-ATG8A with various genetic backgroundsa were incubated in either nitrogen-lacking liquid media for 24 h **(C)** or nitrogen-sufficient liquid media containing AZD8055 for 3 h **(D)** prior to protein extraction. Quantification of relative protein band intensities is shown below. **(E)** Phenotypic analysis for fixed-carbon starvation response. **(F)** A model for FYVE2 and FYVE3 functions in autophagosome dynamics. Columns marked with asterisks represent means that significantly differ from each other, according to the *t*-test. Mean ± S.E.; n = 14 to 16 images **(A**, **B)**, or 4 seedling populations **(C**, **D**, **E)**; *0.01 < p < 0.05; **p < 0.01.

Further genetic analysis, including *atg2 fyve2 fyve3* triple mutants, also confirmed that *FYVE3* is specifically required for the accumulation of GFP-ATG8A puncta in *fyve2* (**Figure 5**). Under N starvation, *atg2 fyve2 fyve3* triple mutants accumulated as many GFP-ATG8A puncta as *atg2*, *atg2 fyve2*, and *atg2 fyve3* mutants (**Figure 5A**), masking the suppressive effects of *fyve3* on *fyve2* mutation. A similar trend was observed when seedlings were treated with AZD8055 (AZD; **Figure 5B**), which induces autophagy by inhibiting Target Of Rapamycin kinase (Soto-Burgos and Bassham, 2017; Dauphinee et al., 2019).

Accumulation of autophagic puncta can result from either induction of autophagy or retarded biogenesis of autophagosomes. Autophagic flux analysis is necessary to distinguish these two possibilities. As *fyve3* alone did not significantly affect autophagic flux (Kim et al., 2022; **Figure S4**), we assessed the effect of *fyve3* on autophagic flux in *atg2* and *fyve2* mutant background. To quantify autophagic flux in different mutants, we used the GFP-ATG8A cleavage assay (Chung et al., 2010; Shin et al., 2014), where anti-GFP immunoblot analysis of seedlings expressing GFP-ATG8A is performed, and autophagic flux is determined from the protein band intensity of free GFP moiety, relative to that of GFP-ATG8A. GFP-ATG8A transgenic seedlings in WT, *atg2*, *fyve2*, and *fyve3*, or their double/triple mutant background were exposed to either N starvation (**Figure 5C**) or AZD (**Figure 5D**) and subjected to GFP-ATG8A cleavage assay. In both conditions, autophagic flux was fully inhibited in any genetic background containing *atg2* and also greatly interfered in *fyve2* (**Figures 5C, D**). In contrast, *fyve2 fyve3* double mutant was indistinguishable from WT and *fyve3*, confirming that *fyve3* suppresses *fyve2* (**Figures 5C, D**).

Finally, the physiological function of *FYVE3* gene expression was tested by phenotypic analysis of *fyve3* single and *fyve2 fyve3* double mutants. Autophagy-deficient mutants are hypersensitive to carbon starvation (Hanaoka et al., 2002; Chung et al., 2010). Our fixed-carbon starvation experiments showed that *fyve2-2* seedlings showed hypersensitivity to light deprivation, whereas *fyve3-1* did not (**Figure 5E**). Importantly, *fyve2-2 fyve3-1* double mutants showed a WT-like sensitivity, demonstrating that the suppression effect of *fyve3* on *fyve2* is physiologically significant.

## Discussion

FYVE3 is a putative PI3P effector involved in autophagy (Chung, 2019), but its function remains unelucidated. In this study, we investigated the role of FYVE3 in plant autophagy. We demonstrated that FYVE3 interacts with ATG8 isoforms (**Figure 1**), associates with ATG8-positive autophagic membranes (**Figure 2**), and is degraded in the vacuole in a manner dependent on core *ATG* genes and PI3K (**Figure 3**). As autophagic flux is not significantly altered by *fyve3* single mutation under N starvation (**Figures 4**, **5**), FYVE3 could be considered an autophagic cargo that does not play a critical role in autophagosome biogenesis. However, this scenario is unlikely, because genetic analysis of *fyve2 fyve3* double mutants indicated that *fyve3* suppressed *fyve2* phenotypes, such as over-accumulation of autophagic puncta, reduction in autophagic flux, and starvation response (**Figures 4**, **5**). This suppression by *fyve3* is in contrast with the hyperaccumulation of autophagic markers in *atg2 fyve3* double mutants, where no suppression was observed. Based on these genetic interactions, we propose that *FYVE3* acts upstream of *FYVE2* but downstream of *ATG2* during autophagosome formation (**Figure 5F**).

The phenotypic consequence of *fyve3* mutation is not evident unless it is combined with *fyve2* mutation, which prevented us and others from identifying its involvement in autophagy (Kim et al., 2022; Zhao et al., 2022). In our previous work (Kim et al., 2022), we employed 48-h nitrogen starvation for autophagic flux assay, whereas 24-h starvation was used for the assay in this study. Nevertheless, we observed a similar suppression in the autophagic flux analysis in which 21-d-old leaves of *fyve2 fyve3* mutants were used (Kim et al., 2022). Zhao et al. (2022) reported that the steady-state level of the autophagic cargo receptor NBR1 was comparable between *fyve2* and *fyve2 fyve3*, but did not directly assess the autophagic flux of *fyve2 fyve3* (i.e., *cfs1 cfs2*) double mutants. As *NBR1* is transcriptionally regulated in an autophagy-independent manner (Jung et al., 2020), further work is needed to investigate how NBR1 is regulated in *fyve2* and *fyve3* mutants.

The biochemical function of FYVE3 on the autophagic membrane is not clear. As autophagic flux is almost completely inhibited by *atg2* but only partially by *fyve2* (Kang et al., 2018; Kim et al., 2022), we postulate two separate autophagic routes, FYVE2-dependent and FYVE2-independent routes (**Figure 5F**). FYVE3 may specifically participate in FYVE2-dependent autophagy. Considering the suppression effect indicating the involvement of FYVE3 in FYVE2-dependent autophagy, the absence of *fyve3* single mutant phenocopying *fyve2* is noteworthy. This can be explained if FYVE3 is a negative regulator of FYVE2-mediated autophagy. For example, FYVE3 may compete with autophagosome biogenesis factors for ATG8 binding. Alternatively, it is possible that FYVE3 both positively mediates the FYVE2-dependent autophagy and negatively regulates the FYVE2-independent route. In the absence of FYVE3, vacuolar delivery of ATG8 through the FYVE2-independent pathway may increase and compensate for a reduced autophagic flux of ATG8 through the FYVE2-dependent autophagy. In the latter model, the interaction of FYVE3 with ATG8 may be responsible for recruiting FYVE2 on expanding phagophores (Kim et al., 2022). Further investigation is needed to reveal the nature of FYVE2-dependent autophagy and a specific role for FYVE3 in this pathway.

In summary, our study uncovers a regulatory module of FYVE2 and FYVE3 and its potential role in autophagosome biogenesis (**Figure 5F**). Our study suggests that FYVE3 participates in FYVE2-dependent autophagy *via* its interaction with PI3P and ATG8 isoforms. Thus, future investigation should ask what regions of FYVE3 are responsible for these interactions, whether FYVE3 is a positive or negative regulator of FYVE2-dependent autophagy, and how core ATG proteins and PI3P collaborate with FYVE2 and FYVE3.

## Data availability statement

The raw data supporting the conclusions of this article will be made available by the authors, without undue reservation.

## Author contributions

JK performed most experiments. JK, HJ, KS, HL and TC developed genetic materials. TC wrote the manuscript with support from JK, HJ and HL. All authors contributed to the article and approved the submitted version.
