# Supplementary material for: The phosphatidylinositol 3-phosphate effector FYVE3 regulates FYVE2-dependent autophagy in Arabidopsis thaliana
Source: Front Plant Sci. 2023 Mar 15;14:1160162. doi: 10.3389/fpls.2023.1160162 (PMC10050702; doi:10.3389/fpls.2023.1160162)
Supplement: Supplementary file 2 [file DataSheet_1.pdf]

Kim et al. Figure S1

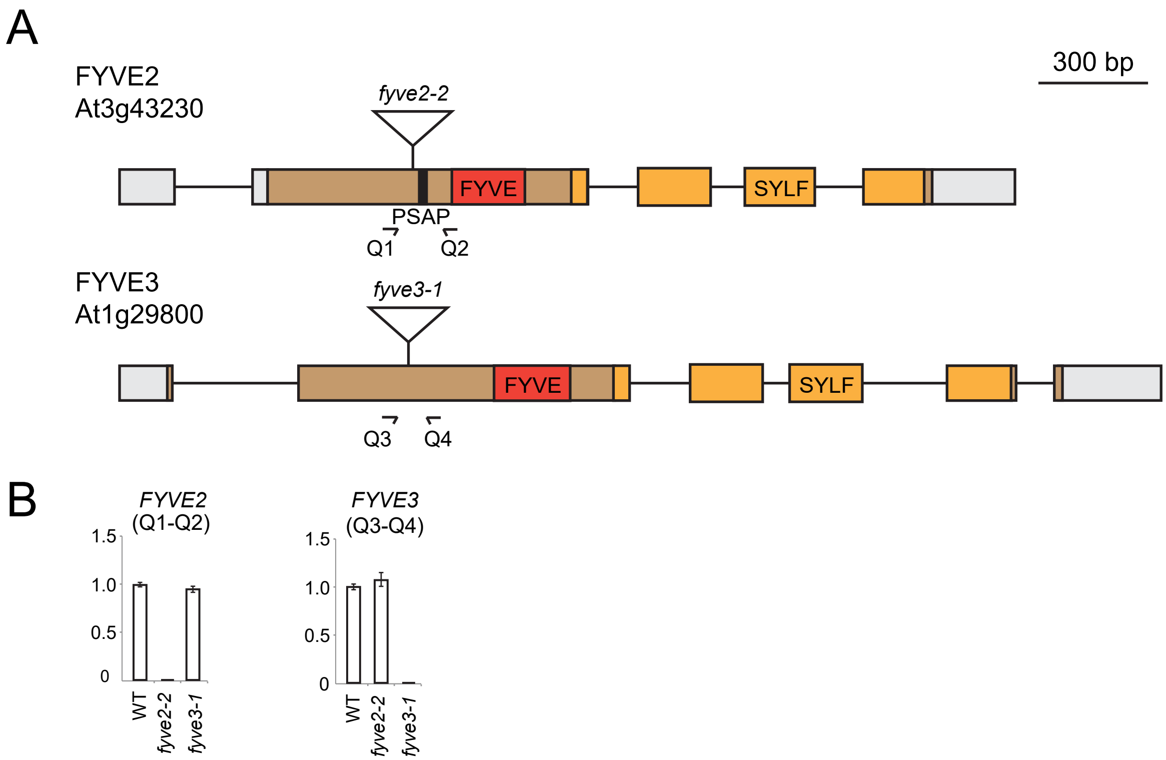

Figure S1. Characterization of *Arabidopsis fyve3* mutants. **A**, Schematic of the *Arabidopsis FYVE2* and *FYVE3* genes. The coding region is illustrated by colored boxes (exons) connected by lines (introns), with FYVE and SYLF domains highlighted in red and orange, respectively. Grey boxes indicate untranslated regions. A vertical line in the second exon of *FYVE2* indicates the PSAP motif required for interaction with VPS23. Open triangles indicate the positions of T-DNA insertions in *fyve2-2* and *fyve3-1*. **B**, Graphs showing RT-PCR data to assess the effect of *fyve2* and *fyve3* on *FYVE2* and *FYVE3* transcript levels. RNA was obtained from WT, *fyve2-2*, and *fyve3-1* seedlings grown on a nitrogen-sufficient solid medium for 10 d. The positions of RT-PCR primers used are indicated by half-arrows in **A**.

Kim et al. Figure S2

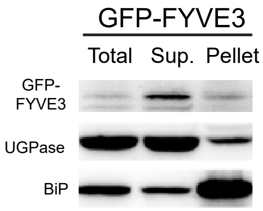

Figure S2. Membrane fractionation analysis showing the solubility of GFP-FYVE3. Immunoblots using antibodies reacting with GFP, UGPase (control for soluble fraction), and ER-resident isoform of heat shock protein 70 (BiP; control for microsomal fraction) are shown.

Kim et al. Figure S3

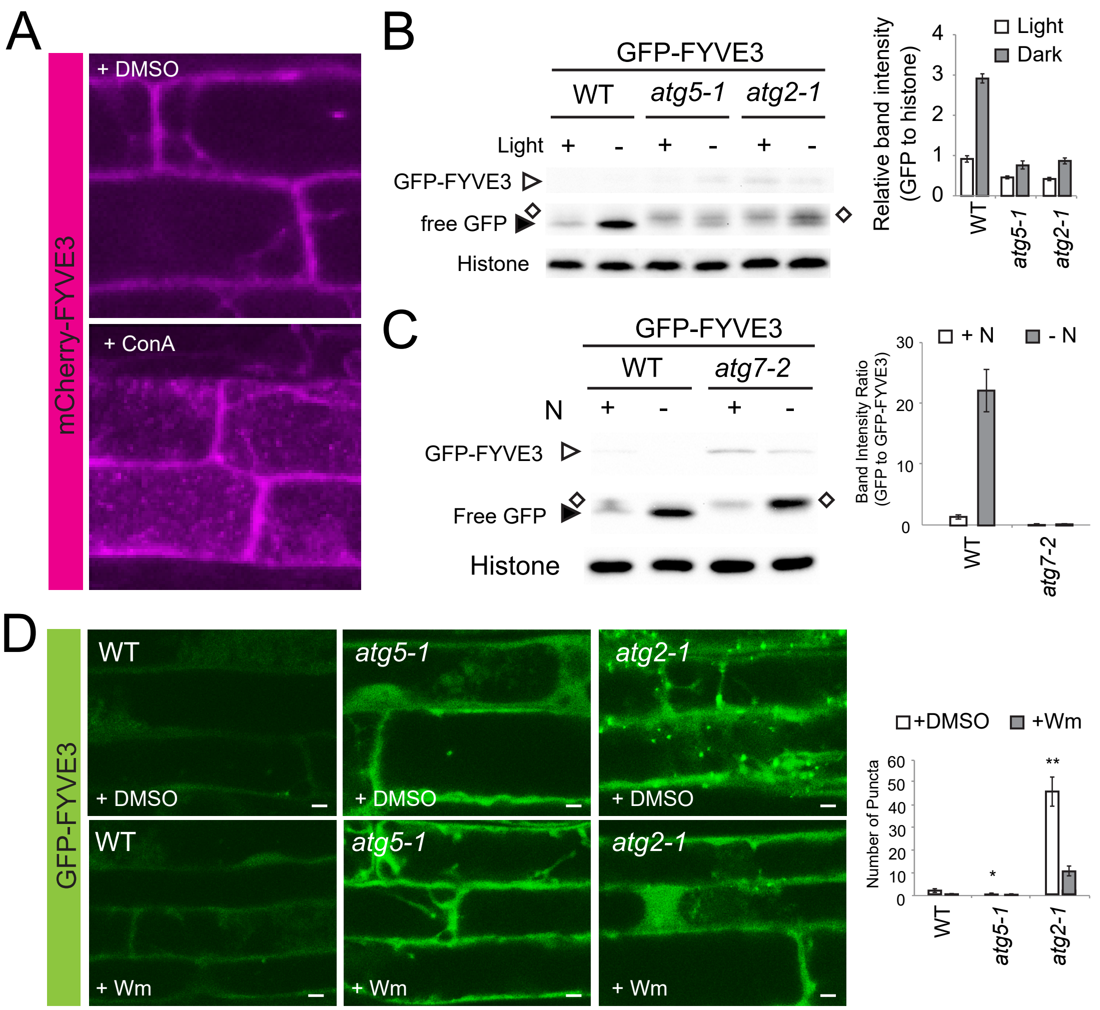

Figure S3. Fluorescent fusions of FYVE3 are degraded in the vacuole via the autophagic route. **A**, Confocal images showing mCherry-FYVE3 puncta in central vacuoles of mature root cells. mCherry-FYVE3 seedlings were incubated in a nitrogen-sufficient liquid medium for 9 d and treated with DMSO or 0.5 µM ConA for 16 h prior to observation. **B**, GFP-FYVE3 cleavage assay to verify that the free GFP band is a product of vacuolar processing. Seedlings expressing GFP-FYVE3 in WT, *atg5-1*, and *atg2-1* background were incubated in a nitrogen-sufficient liquid medium for 8 d and then exposed to light or dark for 24 h before protein extraction. Immunoblots using anti-GFP (upper) and anti-histone H3 (lower; loading control) antibodies are shown. Open diamonds indicate the position of protein bands that migrates slightly slower than free GFP bands (indicated by a solid arrowhead) which are stabilized by light deprivation. The graphs on the right are the quantification of relative intensities of protein bands (mean ± S.E.; n = 4) indicated by the solid arrowhead. **C**, GFP-FYVE3 cleavage assay of nitrogen-starved seedlings. Nine-d-old WT and *atg7-2* seedlings expressing GFP-FYVE3 were incubated in either a nitrogen-sufficient or -lacking medium for 2 d prior to protein extraction. The graphs on the right are the quantification of relative intensities of protein bands (mean ± S.E.; n = 4) indicated by solid and open arrowheads. **D**, Confocal images of mature root cells expressing GFP-FYVE3 in WT, *atg5-1*, and *atg2-1* background. Seedlings were incubated in a nitrogen-sufficient liquid medium for 9 d and treated with DMSO or 30 µM Wm for 1 h, prior to microscopic observation. The graph on the right shows the quantification of GFP-FYVE3 punctum density (number of puncta per 12,656 μm^2; mean ± S.E.; n = 10 to 12). Scale bars = 5 µm.

Kim et al. Figure S4

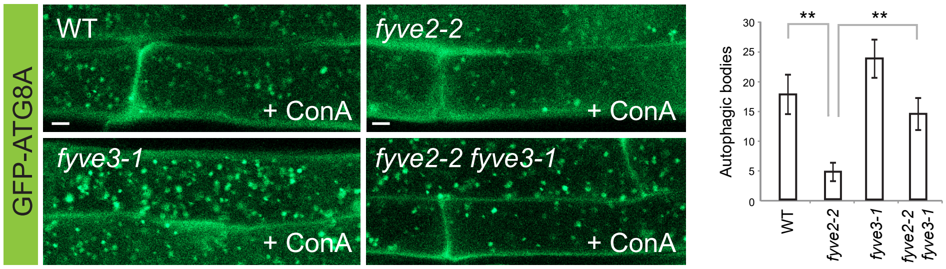

Figure S4. Autophagic flux is not impaired in *fyve3*. Confocal microscopic images of root cells accumulating autophagic bodies. Transgenic seedlings expressing the autophagic marker GFP-ATG8A were incubated in nitrogen-lacking liquid medium for 48 h and treated with 0.5 µM ConA for 16 h prior to microscopy. The graph on the right shows the quantification of GFP-ATG8A punctum density (number of puncta per 1,000 µm^2; mean ± S.E.; n = 14 to 17). Scale bars = 5 µm.

Kim et al. Figure S5

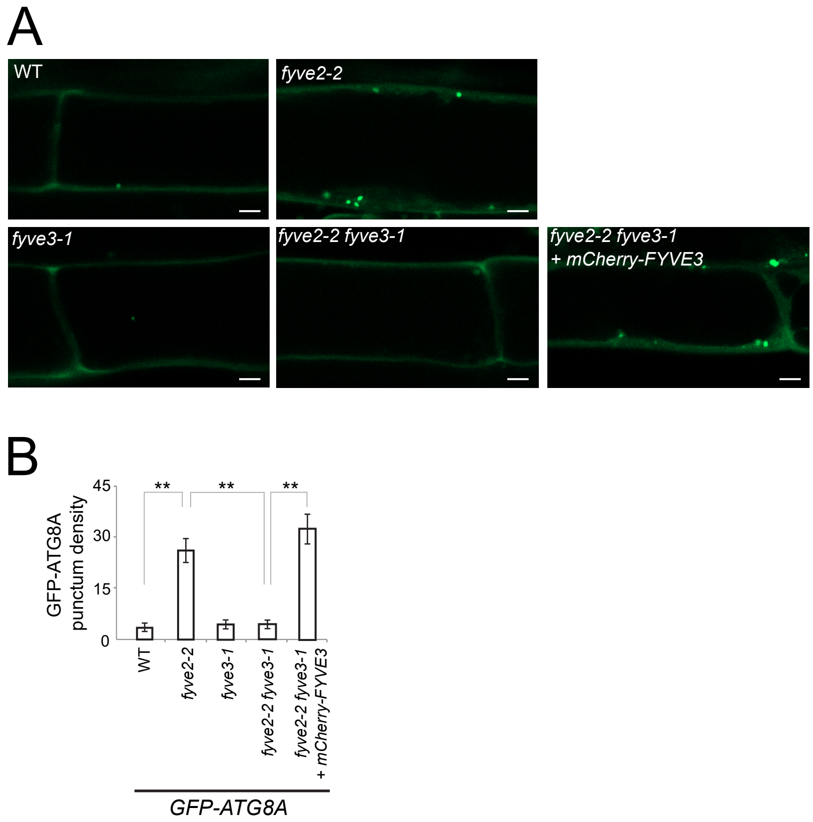

Figure S5. Overexpression of *FYVE3* transgene complements the *fyve3-1* phenotypes. **A**, Confocal microscopic images of GFP-ATG8A-expressing root cells. Seedlings with the indicated genotypes were incubated in a nitrogen-lacking liquid medium for 2 d, prior to microscopic observation. Scale bars = 5 µm. **B**, Graph showing GFP-ATG8A punctum density per 25,514 μm^2. Autophagic puncta were counted from mature root cells of seedlings expressing GFP-ATG8A. Columns marked with asterisks represent means that significantly differ from each other, according to *t*-test. Mean ± S.E.; n = 14 to 16 images; **, $p < 0.01$.
